# The decisions and processes involved in a systematic search strategy: a hierarchical framework

**DOI:** 10.5195/jmla.2021.1086

**Published:** 2021-04-01

**Authors:** Justin Michael Clark, Elaine Beller, Paul Glasziou, Sharon Sanders

**Affiliations:** 1 jclark@bond.edu.au, Institute for Evidence-Based Healthcare, Bond University, Robina, Queensland, Australia; 2 ebeller@bond.edu.au, Institute for Evidence-Based Healthcare, Bond University, Robina, Queensland, Australia; 3 pglaszio@bond.edu.au, Institute for Evidence-Based Healthcare, Bond University, Robina, Queensland, Australia; 4 ssanders@bond.edu.au, Institute for Evidence-Based Healthcare, Bond University, Robina, Queensland, Australia

**Keywords:** systematic reviews, systematic searching, evidence identification, evidence synthesis

## Abstract

**Objective:**

The decisions and processes that may compose a systematic search strategy have not been formally identified and categorized. This study aimed to (1) identify all decisions that could be made and processes that could be used in a systematic search strategy and (2) create a hierarchical framework of those decisions and processes.

**Methods:**

The literature was searched for documents or guides on conducting a literature search for a systematic review or other evidence synthesis. The decisions or processes for locating studies were extracted from eligible documents and categorized into a structured hierarchical framework. Feedback from experts was sought to revise the framework. The framework was revised iteratively and tested using recently published literature on systematic searching.

**Results:**

Guidance documents were identified from expert organizations and a search of the literature and Internet. Data were extracted from 74 eligible documents to form the initial framework. The framework was revised based on feedback from 9 search experts and further review and testing by the authors. The hierarchical framework consists of 119 decisions or processes sorted into 17 categories and arranged under 5 topics. These topics are “Skill of the searcher,” “Selecting information to identify,” “Searching the literature electronically,” “Other ways to identify studies,” and “Updating the systematic review.”

**Conclusions:**

The work identifies and classifies the decisions and processes used in systematic searching. Future work can now focus on assessing and prioritizing research on the best methods for successfully identifying all eligible studies for a systematic review.

## INTRODUCTION

A key factor affecting the quality of evidence syntheses, such as systematic reviews (SRs), is the inclusion of a comprehensive, reproducible, and well-conducted systematic search strategy [1]. An inadequate or poorly implemented search can miss relevant studies (i.e., poor recall) and impact the findings of the SR [2]. It may also increase the number of irrelevant articles that need to be screened (i.e., poor precision), adding to the time and resources required to conduct the SR.

Many guides exist to help conduct a systematic search [3–7], most of which recommend a standard approach that entails searching several key health databases using two or three different search concepts with large numbers of synonyms for each concept. The search results are then supplemented by a check of the reference lists of included studies. The focus is on achieving high recall: finding all the relevant studies addressing the review question. Precision—the number of relevant versus irrelevant studies found—and the time required to conduct the systematic search and screening process are secondary concerns. The resource burden of ensuring all relevant articles are found (100% recall) is high. The number of irrelevant studies found varies substantially across searches but can number in the tens of thousands [8, 9]. The time burden is also high, with the search, retrieval, screening, and extracting tasks being the most time consuming in an SR [10]. Although times vary considerably, designing and running a systematic search can take more than fifty hours [9]. Thus, recent research has been conducted to attempt to find alternate, or enhanced, ways of conducting systematic searches by modifying workflows or automating some of the processes [9, 11, 12].

To facilitate research aimed at improving and streamlining systematic searches, it is necessary to identify the decisions made and processes used in their design and conduct. Collation of these decisions and processes into a framework will support the evaluation or assessment of the evidence base of standard and new approaches and will assist in the development of a common vocabulary around systematic searching. Although some decisions and processes were previously identified, these have either focused on a single decision or process (e.g., peer review of database search strings) or provided a high-level overview of the searching process without detailing what is involved in each step (e.g., suggesting searching bibliographic databases but not describing in detail how to develop the search strings for those databases) [13–16]. Therefore, the authors aimed to (1) identify all the decisions or processes that could be used in a systematic search and (2) collate related decision and processes together into a hierarchical framework.

## METHODS

We developed a hierarchical framework in four steps:

1. Identify decisions and processes from documents on searching.
2. Create a hierarchical framework of decisions and processes involved in a systematic search.
3. Revise the hierarchical framework based on feedback from experts.
4. Test the hierarchical framework on recently published documents to determine if any decisions or processes were missing.

We defined a systematic search decision or process as a “decision or process made or done to identify documents for review by teams for inclusion in an evidence synthesis.” This purposely excluded tasks that may be done during the design and execution of a systematic search but that are not part of the search itself (e.g., refining the SR question or deduplicating the search results).

Although the hierarchal framework was developed iteratively, some initial parameters were used to guide its development, including: (1) the framework should cover all decisions and processes involved in designing and running a systematic search; (2) multiple decisions or processes should be able to be assigned to an individual document; and (3) each decision or process in the framework should be mutually exclusive.

### Step 1: Identify decisions and processes from documents on searching

We identified documents on conducting a systematic search by: (1) checking the websites of organizations that specialize in conducting SRs (i.e., Cochrane, Campbell Collaboration, Joanna Briggs Institute, Centre for Evidence-Based Medicine, National Institute for Health and Care Excellence, and Centre for Reviews and Dissemination [3–7]); (2) performing a search of the literature in PubMed; Library, Information Science and Technology Abstracts (LISTA); and Google Scholar on March 8, 2018, (search strings for each database available in supplemental Appendix A); and (3) performing a Google search for Internet-based help guides on March 8, 2018 (search string available in supplemental Appendix A). No date, language, or publication restrictions were imposed.

We included journal articles, web guides, book chapters, or other documents that provided advice, guidance, or recommendations on how to conduct a systematic search. We excluded those that provided advice on how to use tools or databases to retrieve studies (e.g., web guide on how to use the PubMed interface). We also excluded editorials and commentaries as this type of opinion-based work was to be obtained through step 3, feedback from experts.

The search results were initially screened by a single author for eligibility, creating a pool of potentially eligible documents. All documents in this pool were independently screened by two authors, and any disagreements were resolved through discussion.

Data from each included document were entered into a data extraction form (supplemental Appendix B). The data extracted included: type of document (e.g., journal article, website, web guide, book chapter); main purpose of the document (e.g., an overview of how to conduct a SR); the type of evidence synthesis that was the focus of the document (e.g., SR, clinical practice guideline [CPG], health technology assessment [HTA], or literature review [LR]); and the systematic search decisions or processes discussed in the document (e.g., using word frequency analysis to design a systematic search strategy).

Due to overlap and duplication of data in the included documents, data were only extracted from documents if they contained at least one new decision or process not already extracted.

### Step 2: Create a hierarchical framework of decisions and processes involved in a systematic search

After data extraction, each decision or process was sorted into topics and categories. Related decisions and processes were grouped into categories, and related categories were grouped into topics. This created a structured entry for each decision or process:

3: Searching the literature electronically (Topic)
3.3: Selecting search words and terms (Category)
3.3.5: Obtaining search words from experts (Decision or process)

Decisions or processes that were extracted but upon closer examination turned out to be the same were merged. For example, if an extracted decision or process stated, “use AND or OR” and another stated “use Boolean operators,” these were combined. The selection of wording used in the framework was made through discussion and was based upon judgments of terminology, clarity, brevity, and usefulness. A single author created an initial version of the framework, which was then reviewed by the other authors during several meetings to refine the draft framework through discussion.

### Step 3: Revise the hierarchical framework based on feedback from experts

To ensure all decisions and processes were identified and categorized appropriately, feedback was sought from experts in literature searching. An email was sent to the Cochrane Information Specialists (CIS) email list that outlined the project and asked for feedback; nine people responded. Changes to the framework based on this feedback were initially made by a single author. All authors then met to discuss the changes and agree upon a final hierarchical framework.

### Step 4: Test the hierarchical framework on recently published documents to determine if any decisions or processes were missing

The framework was tested by identifying recently published articles on systematic searching and attempting to categorize the processes and decisions mentioned in the articles. Recent articles were identified by updating the original search in PubMed and LISTA and limiting the results to those published in 2019. These search results were screened to identify any articles on the topic of systematic searching. One author determined the primary focus of the document and checked it against the hierarchical framework. If any documents could not be assigned an existing decision or process, this led to additions to the hierarchical framework.

## RESULTS

### Results of searches for documents on conducting a systematic search

**Figure 1:**
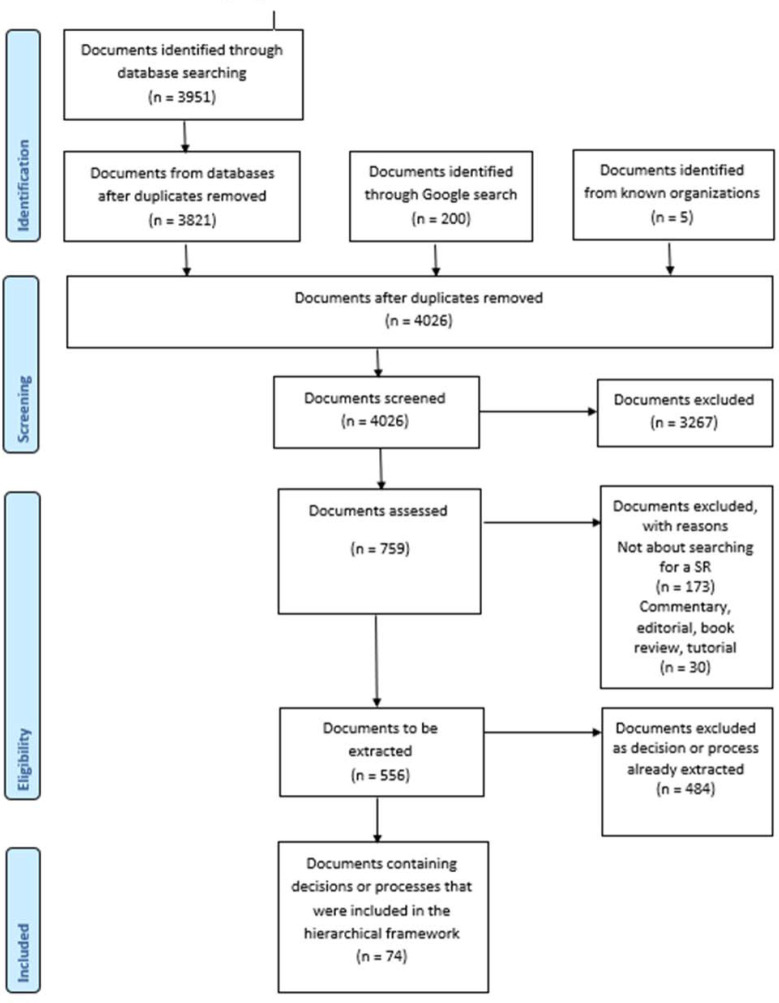
Flowchart of search for documents reporting decisions or processes for conducting a systematic search

Five documents were identified from organizations known to work in the area of SRs [3–7]. All decisions and processes in these documents were extracted. A search of PubMed, LISTA, and Google Scholar identified 3,951 documents. After removal of duplicates, 3,821 unique documents remained. A Google search was also performed, with the first 200 results being screened. After screening, 556 documents were assessed as potentially including decisions or processes that could contribute to the framework. Data were extracted from 74 documents (supplemental Appendix C). Data were not extracted from the remaining 484 documents, as the decision or process had been extracted previously. Figure 1 provides the adapted PRISMA flow diagram [17].

### Initial design of the hierarchical framework

After data extraction and during the grouping of decisions and processes, we determined that a three-level framework would be most suitable for our purpose. A further refinement was to include decisions or processes that could be made or done but not specific recommendations for their implementation (e.g., the decision to search clinical trial registries would be in the framework, but the decision of which trial registries to search would not). We also developed scope notes in the form of short explanatory sentences that described the meaning of the topic, category, and decision or process.

### Feedback from experts on the hierarchical framework

Feedback from experts was primarily focused on rewording of the headings and scope notes to improve clarity. No major revisions of the structure of the framework were suggested. Most feedback revolved around categories, decisions or processes, and scope notes. As a result of this feedback, no topics were added or removed. Two categories and ten decisions or processes were added. One category was removed along with its accompanying four decisions or processes. Three decisions or processes were merged due to their similarity. Just over half of the scope notes were edited, with examples added to improve their clarity. Most changes involved separating existing decisions and processes (e.g., “Searching for grey literature” was changed to “Searching for government reports,” “Searching for dissertations,” “Searching for unpublished data,” etc.; “Refining a search string” was expanded to “Refining to improve recall” and “Refining to improve precision”). Only two additions were made from the experts’ personal knowledge that was not reflected in the included documents: “Excluding predatory journals” and “Using word frequency.”

### Final revision of the hierarchical framework

Revision of the framework was an iterative process, taking place over multiple meetings. After the feedback from experts was incorporated, the authors met to revise the layout and wording of the framework. After six such meetings held over approximately three months, the framework was finalized.

### Testing of the hierarchical framework

The updated search retrieved 68 documents. After screening for relevance, 48 were excluded, and 20 were included (supplemental Appendix D). The primary focus of all 20 included documents could be mapped to an existing decision or process in the hierarchical framework. Over half of the documents (n=11, 55%) were about the decision or process “Using validated search filters.” Most documents (n=17, 85%) were covered by the topic “Searching the literature electronically,” whereas few were covered by the topics “Selecting information to identify” (n=2, 10%) or “Other ways to identify studies” (n=1, 5%). None were covered by the topics “Skill of the searcher” or “Updating the systematic review.” Individual mapping of each study can be found in supplemental Appendix E.

### Hierarchical framework of decisions and processes involved in a systematic search

The final hierarchical framework consists of five topics, each with multiple categories, with each category consisting of multiple decisions or process (Table 1). The specific decisions and processes within each category are shown in Tables 2–6.

**Table 1:** Topics, categories, and number of decisions or processes included in the hierarchical framework

Topic	Category	No. of decisions or processes
1: Skill of the searcher (Table 2)	1.1: Searcher role	5
	1.2: Searcher experience	5
2: Selecting information to identify (Table 3)	2.1: Selecting publication formats	13
	2.2: Selecting electronic sources to search	9
3: Searching the literature electronically (Table 4)	3.1: Obtaining a development article set	4
	3.2: Conceptualizing the search string	16
	3.3: Selecting search words and terms	9
	3.4: Using database search commands	12
	3.5: Refining a search string	9
	3.6: Running a search string	5
4: Other ways to identify studies (Table 5)	4.1: Using databases similarity feature	1
	4.2: Contacting people	7
	4.3: Citation analysis	8
	4.4: Hand searching	5
	4.5: Non-systematic study identification	1
5: Updating the systematic review (Table 6)	5.1: Updating the systematic search	5
	5.2: Running the updated systematic search	5

**Table 2:** Decisions or processes related to “Skill of the searcher”

1: Skill of the searcher	
1.1: Searcher role	1.2: Searcher experience
1.1.1: Authors conducting the search	1.2.1: Systematic review experience
1.1.2: Cochrane information specialist conducting the search	1.2.2: Experience with the topic
1.1.3: Information specialist conducting the search	1.2.3: Systematic searching experience
1.1.4: Health librarian conducting the search	1.2.4: General literature search experience
1.1.5: General librarian conducting the search	1.2.5: Training in searching

**Table 3:** Decisions or processes related to “Selecting information to identify”

2: Selecting information to identify	
2.1: Selecting publication formats	2.2: Selecting electronic sources to search
2.1.1: Searching for journal articles	2.2.1: Searching bibliographic databases
2.1.2: Searching for ongoing studies	2.2.2: Searching full-text databases
2.1.3: Searching for nongovernment reports	2.2.3: Searching specialized registers
2.1.4: Searching for books or book chapters	2.2.4: Searching Google Scholar
2.1.5: Searching for conference proceedings	2.2.5: Searching trial registries
2.1.6: Searching for dissertations	2.2.6: Searching the Internet
2.1.7: Searching for correspondence	2.2.7: Searching specific websites
2.1.8: Searching for electronic publications ahead of print	2.2.8: Searching social media platforms
2.1.9: Searching for language-specific information	2.2.9: Costs of searching
2.1.10: Searching for government reports	
2.1.11: Searching for unpublished work	
2.1.12: Searching for errata or corrections	
2.1.13: Excluding predatory journals	

**Table 4:** Decisions or processes related to “Searching the literature electronically”

3: Searching the literature electronically		
3.1: Obtaining a development article set	3.2: Conceptualizing the search string	3.3: Selecting search words and terms
3.1.1: Obtaining a development set from systematic reviews	3.2.1: Selecting search concepts	3.3.1: Selecting index terms
3.1.2: Obtaining a development set from experts	3.2.2: Selecting concepts from the systematic reviews patient, intervention, comparison, outcome (PICO)	3.3.2: Searcher selecting search words
3.1.3: Obtaining a development set from a scoping search	3.2.3: Selecting concepts using a structured format	3.3.3: Selecting search words from a development set
3.1.4: Obtaining a development set from a citation analysis	3.2.4: Using broad or focused concepts	3.3.4: Selecting search words from word frequency analysis
	3.2.5: Importance of recall	3.3.5: Obtaining search words from experts
	3.2.6: Importance of precision	3.3.6: Selecting search words from similar articles
	3.2.7: Searching the full text	3.3.7: Using synonyms
	3.2.8: Selecting a user interface	3.3.8: Using alternate spellings
	3.2.9: Database used for designing primary search string	3.3.9: Using words in other languages
	3.2.10: Searching for older studies	
	3.2.11: Ordering of search words	
	3.2.12: Using “AND” or “OR”	
	3.2.13: Using “Adjacency”	
	3.2.14: Using “NOT”	
	3.2.15: Using validated search filters	
	3.2.16: Using non-validated search strings	
3.4: Using database search commands	3.5: Refining a search string	3.6: Running a search string
3.4.1: Exploding index terms	3.5.1: Refining to improve recall	3.6.1: Single line searching
3.4.2: Focusing index terms	3.5.2: Refining to improving precision	3.6.2: Line-by-line searching
3.4.3: Using search words and index terms	3.5.3: Using validation articles	3.6.3: Block searching
3.4.4: Using search words alone	3.5.4: Updating search words and index terms	3.6.4: Modifying for other databases
3.4.5: Using index terms alone	3.5.5: Using words in other languages	3.6.5: Modifying for nonbibliographic databases
3.4.6: Using subheadings	3.5.6: Discussing the search string with experts	
3.4.7: Using predefined limits	3.5.7: Peer reviewing the search string	
3.4.8: Using wildcards and truncation	3.5.8: Using spell checking on the search string	
3.4.9: Using phrase searching	3.5.9: Finalizing the search string	
3.4.10: Searching fields		
3.4.11: Using term mapping		
3.4.12: Using word frequency		

**Table 5:** Decisions or processes related to “Other methods to identify relevant studies”

4: Other ways to identify studies		
4.1: Using databases similarity feature	4.2: Contacting people	4.3: Citation analysis
4.1.1: Using a related articles feature	4.2.1: Contacting experts	4.3.1: Selecting a citation database
	4.2.2: Contacting funders	4.3.2: Conducting a forward citation analysis
	4.2.3: Contacting authors of included studies	4.3.3: Conducting a backward citation analysis
	4.2.4: Contacting manufacturers	4.3.4: Conducting a co-citing articles analysis
	4.2.5: Contacting regulatory agencies	4.3.5: Conducting a co-cited articles analysis
	4.2.6: Contacting specialist organisations	4.3.6: Manually checking reference lists
	4.2.7: Soliciting eligible studies	4.3.7: Checking other systematic reviews
		4.3.8: Iterative citation analysis
4.4: Hand searching	4.5: Non-systematic study identification	
4.4.1: Hand searching journals	4.5.1: Browsing the literature	
4.4.2: Hand searching websites		
4.4.3: Hand searching conference proceedings		
4.4.4: Hand searching bookshelves and filing cabinets		
4.4.5: Hand searching personal collections		

**Table 6:** Decisions or processes related to “Updating searches”

5: Updating the systematic review	
5.1: Updating the systematic search	5.2: Running the updated systematic search
5.1.1: Receiving table of contents alerts	5.2.1: Modifying original sources
5.1.2: Receiving search alerts	5.2.2: Using search alerts
5.1.3: Periodically rerunning searches	5.2.3: Using date limitations
5.1.4: Surveying the literature	5.2.4: Revising the search string
5.1.5: Monitoring eligible registered trials	5.2.5: Searching for retractions

The full hierarchical framework with scope notes for each entry can be found online [18] and in supplemental Appendix F.

## DISCUSSION

We identified many decisions made during or processes used to conduct a systematic search for an evidence synthesis, which we organized into a hierarchical framework using an iterative process of searching, feedback, testing, and discussion. This hierarchical framework can be used to quantify the amount and quality of evidence that supports the use of each identified decision or process. Additional benefits of the framework could be to identify the decisions and processes that could benefit from greater efficiency—in terms of recall, precision, and time—and use them to modify or enhance the current “standard” way of searching. Another future benefit would be to begin the standardization of terminology used to discuss systematic search strategies.

Other researchers have identified decisions or processes used in systematic searches. None were sufficiently comprehensive or detailed for our requirements. For example, the PRESS checklist highlighted many components of a systematic search that can be peer reviewed to improve its quality, focused on the search of electronic databases, and covered the creation of search strings in a broad way [13]. A review article covered eight broad themes that were considered to be important when searching for studies [19] and was later updated but still focused on broad themes [20]. Another study that defined the process of literature searching identified eight broad themes [21], whereas a similar study of supplementary search strategies identified five alternate methods [16]. That study also only gave broad definitions, which we called topics or categories in our framework [16]. Our study appears to be the first to identify and group together in detail all the decisions and processes that could be used to design and conduct a systematic search strategy.

Summaries of evidence or studies on systematic search strategies do exist, although formal assessment of their quality does not appear to have been done. Two reviews collated research studies but did not assess them [16, 20]. A web-based summary platform sorted articles into categories but also did not provide a formal assessment of their quality [22]. In addition, the PRESS checklist, although it was a comprehensive document of research on peer reviewing systematic searches, did not formally assess the quality of evidence; rather, it utilized a web-based survey of experts and a consensus forum to add to or adjust its recommendations [23]. This highlights the benefits of the current work as a precursor to assessing the evidence.

This hierarchical framework lends itself to the creation of standardized terminology for research on systematic searching. The terminology used to discuss systematic searching is varied. The current, standard approach to searching the literature can be referred to in multiple ways, such as the standard approach, the conventional approach, the traditional approach, or the conceptual approach [24, 25]. Some of the most basic decisions or processes of systematic searching are not defined; for instance, whether to use the terms “keyword” or “free text” to describe searching for words that appear in documents [3]. Supplementary methods also suffer from this problem, as checking reference lists or citing articles can be referred to as snowballing, pearl growing, reference checking, or citation analysis [16, 20].

In most cases, designing and running a systematic search requires many hours of work. A survey of 105 librarians who recently worked on an SR showed that the average duration for all tasks was 30.7 hours, with a range of 2 to 219 hours. For those tasks covered by the framework—discussing, designing, and running the search—the average was 17.7 hours [26]. Despite time being an important factor in searching, we decided to exclude it from the framework because the time spent searching is rarely, if ever, a decision that is explicitly made. Rather, time spent searching depends upon every other decision made or process done. This was reflected in the literature, because although mention is made of “within resource limits” or “appropriate amount of time,” in practice this never happens.

In our experience, a good search that finds as much relevant evidence as possible takes as long as it takes. Good searches for simple reviews can be quick; good searches for complex reviews take a long time. Never, in our experience, has any SR team ever said they were happy with a bad search that may miss large amounts of relevant evidence. Despite this, recent advances in SR methodology, such as the 2weekSR method [27] and search automation tools such as the Polyglot Search Translator [11], show that time is becoming an important topic for systematic search specialists. Therefore, if the framework is updated in the future, time spent searching could be an explicit decision that is made and, therefore, would be added to the framework. For example, in the 2weekSR method, the search needs to be completed by the end of day one; therefore, using time-saving measures, such as automation tools, is a decision that would need to be explicitly made.

This hierarchical framework can be a foundational piece of work for future research projects. For example, standardization of terminology in systematic searching is needed, and this framework starts that standardization process. If a future update of this framework occurs, then this standardization could continue. Preferably, this future update would involve search experts from a broader, more international pool than those used in this version, which would increase the chances of the framework terminology being adopted. Currently, there is a lack of certainty around which decisions and processes are effective, which make little impact, and which make searches less efficient. Identifying evidence of the effectiveness of each decision and process should also be easier with standardization. This would allow a review of the current evidence and the grouping of that evidence by decision or process, which is a research project currently underway by our author team.

Further work could look at the applicability of the framework to systematic searching in fields outside of health care, such as economics. Finally, testing each decision or process individually has now become more feasible. A major issue with research evaluating systematic searching is that it is hard to remove the confounding of the expertise of the searchers. With this framework, it should now be easier to isolate each decision or process and test a search with and without them, which should help with removing, or minimizing, the confounders that interfere with research on systematic searching. We are currently designing a research project to explore the feasibility of accomplishing this. Thus, this framework lays the foundation for stronger, more impactful research into systematic searching. Our hope is that other groups will use it to help plan and report their research in a standardized and targeted way.

The strengths of this study are that the decisions and processes were identified from a comprehensive search of the existing literature (to ensure that any decisions or processes that were not known or utilized by experts would be identified) and through consultation with search experts. The framework was developed through an iterative process incorporating feedback from experts and was tested using recent studies that were not used to inform the development of the framework. A potential weakness of this study is that feedback was obtained from experts on health care SRs and, thus, may have missed decisions or processes used by experts who work in other fields. Also, feedback on the framework was sought from the small Cochrane Information Specialists email list, which could have biased the framework to the decisions and processes used on Cochrane reviews rather than SRs in general.

Systematic searches for evidence syntheses involve many decisions and processes. Our work identifies and classifies these decisions and processes. Future work can now focus on assessing and prioritizing research on the best methods for successfully identifying all eligible studies for a SR.

## Data Availability

Data associated with this article are available via the Bond University Library https://research.bond.edu.au/en/datasets/the-decisions-and-processes-involved-in-a-systematic-search-strat [18].

## SUPPLEMENTAL FILES

**Appendix A:** Search strategies to identify decisions or processes used to conduct a systematic search strategy

**Appendix B:** Data extraction form, with examples

**Appendix C:** Documents used to create the hierarchical framework

**Appendix D:** Studies used to test the hierarchical framework

**Appendix E:** Decisions or processes of studies on searching identified in 2019

**Appendix F:** Hierarchical framework of decisions or processes that can be used to conduct a systematic search with scope notes
