# Supplementary material for: The decisions and processes involved in a systematic search strategy: a hierarchical framework
Source: J Med Libr Assoc. 2021 Apr 1;109(2):201–11. doi: 10.5195/jmla.2021.1086 (PMC8270345; doi:10.5195/jmla.2021.1086)
Supplement: Supplementary file 1 — Appendix A: Search strategies to identify decisions or processes used to conduct a systematic search strategy [file jmla-109-2-201-s01.docx]

# The decisions and processes involved in a systematic search strategy: a hierarchical framework

## Justin Michael Clark; Elaine Beller; Paul Glasziou; Sharon Sanders

### APPENDIX A

### Search strategies to identify decisions or processes used to conduct a systematic search strategy

**PubMed search date: March 8, 2018**

(Search[ti] OR Searches[ti] OR Searching[ti] OR Searchers[ti] OR Retrieval[ti] OR Retrieve[ti] OR Retrieves[ti] OR Retrieving[ti] OR Identify[ti] OR Identification[ti] OR Identifying[ti] OR Locate[ti] OR Locates[ti] OR Locating[ti] OR Find[ti] OR Finds[ti] OR Finding[ti])

AND

("Information Storage and Retrieval"[Mesh] OR "Review Literature as Topic"[Mesh] OR "Search engine"[Mesh] OR "Abstracting and Indexing as Topic"[Mesh] OR "Subject Headings"[Mesh] OR "Medical Subject Headings"[Mesh] OR "Vocabulary, Controlled"[Mesh] OR "Databases as Topic"[Mesh] OR "Study identification" [tiab] OR "Systematic search strategy" [tiab] OR "Search strategies" [tiab] OR "Retrieval strategies" [tiab] OR "Retrieval strategies" [tiab] OR "Search methods" [tiab])

AND

(MEDLINE[Mesh] OR PubMed[Mesh] OR Databases, Bibliographic[Mesh] OR Review[tiab] OR Reviews[tiab] OR "Clinical practice guideline" [tiab] OR "Clinical practice guidelines" [tiab] OR CPG[tiab] OR CPGs[tiab] OR "Health technology assessment" [tiab] OR "Health technology assessments" [tiab] OR HTA[tiab] OR HTAs[tiab] OR "Meta analysis" OR "Meta-analysis" [tiab] OR Cochrane[tiab] OR PubMed[tiab] OR Medline[tiab] OR Embase[tiab])

NOT

("Data Compression"[Mesh] OR "Molecular Sequence Data"[Mesh] OR molecular[tiab] OR DNA[tiab] OR RNA[tiab] OR computational[tiab] OR genome[tiab] OR proteomic[tiab] OR genomic[tiab] OR Metagenomic[tiab] OR spectra[tiab] OR spectrography[tiab] OR chromatography[tiab] OR "Cochrane Database Syst Rev"[jour])

**Library, Information Science & Technology Abstracts (LISTA) search date: March 8, 2018**

No limits, Includes: Academic Journals, Magazines, Reviews, Trade Publications, Books, Newspapers

(TI Search OR TI Searches OR TI Searching OR TI Searchers OR TI Retrieval OR TI Retrieve OR TI Retrieves OR TI Retrieving OR TI Identify OR TI Identification OR TI Identifying OR TI Locate OR TI Locates OR TI Locating OR TI Find OR TI Finds OR TI Finding)

AND

(DE "INFORMATION retrieval" OR DE "ABSTRACTING" OR DE "AUTHORITY files (Information retrieval)" OR DE "BAR codes" OR DE "BIBLIOGRAPHICAL searching" OR DE "BOOLEAN searching (Online information retrieval)" OR DE "CATALOGING" OR DE "CROSS references (Information retrieval)" OR DE "CROSS-language information retrieval" OR DE "ELECTRONIC information resource searching" OR DE "FALLOUT (Information retrieval)" OR DE "FILTERING of information" OR DE "GENOMIC information retrieval" OR DE "IMAGE retrieval" OR DE "INDEXING" OR DE "INFORMATION foraging theory" OR DE "INFORMATION services" OR DE "INFORMATION-seeking strategies" OR DE "INTERNET searching" OR DE "INVERSE document frequency" OR DE "LATENT semantic analysis" OR DE "LIBRARY research" OR DE "LINK resolvers" OR DE "METADATA harvesting" OR DE "PRECISION (Information retrieval)" OR DE "RECALL (Information retrieval)" OR DE "RELEVANCE ranking (Information science)" OR DE "SOUND retrieval" OR DE "STATISTICAL matching" OR DE "STOP words" OR DE "TEXT mining (Information retrieval)" OR DE "VECTOR-space models (Information retrieval)" OR TI "Study identification" OR AB "Study identification" OR TI "Systematic search strategy" OR AB "Systematic search strategy" OR TI "Search strategies" OR AB "Search strategies" OR TI "Retrieval strategies" OR AB "Retrieval strategies" OR TI "Retrieval strategies" OR AB "Retrieval strategies" OR TI "Search methods" OR AB "Search methods")

AND

(DE "MEDLINE" OR DE "DATABASES" OR DE "ONLINE databases" OR TI Review OR AB Review OR TI Reviews OR AB Reviews OR TI "Clinical practice guideline" OR AB "Clinical practice guideline" OR TI "Clinical practice guidelines" OR AB "Clinical practice guidelines" OR TI CPG OR AB CPG OR TI CPGs OR AB CPGs OR TI "Health technology assessment" OR AB "Health technology assessment" OR TI "Health technology assessments" OR AB "Health technology assessments" OR TI HTA OR AB HTA OR TI HTAs OR AB HTAs OR "Meta analysis" OR TI Meta-analysis OR AB Meta-analysis OR TI Cochrane OR AB Cochrane OR TI PubMed OR AB PubMed OR TI Medline OR AB Medline OR TI Embase OR AB Embase)

**Google Scholar search date: March 14, 2018**

(allintitle: Search OR Searches OR Searching OR Searchers OR Retrieval OR Retrieves OR Retrieving OR Identification) AND (Review OR Reviews OR "Meta analysis" OR "Meta analysis" OR Cochrane OR PubMed OR Medline OR Embase)

**Google search date: March 14, 2018**

How to search for studies for a systematic review

How to search for articles for a systematic review

How to locate for studies for a systematic review

How to locate for articles for a systematic review

How to retrieve for studies for a systematic review

How to retrieve for articles for a systematic review
