# Supplementary material for: The decisions and processes involved in a systematic search strategy: a hierarchical framework
Source: J Med Libr Assoc. 2021 Apr 1;109(2):201–11. doi: 10.5195/jmla.2021.1086 (PMC8270345; doi:10.5195/jmla.2021.1086)
Supplement: Supplementary file 2 — Appendix B: Data extraction form, with examples [file jmla-109-2-201-s02.docx]

# The decisions and processes involved in a systematic search strategy: a hierarchical framework

## Justin Michael Clark; Elaine Beller; Paul Glasziou; Sharon Sanders

### APPENDIX B

### Data extraction form, with examples

| **Study ID** | **Document type (journal article, website, web guide, book chapter)** | **Main purpose of the search method described in the document (if applicable)** | **Purpose description (copied and pasted from article)** | **Study type document about (SR, HTA, CPG, LR)** | **Broad article topic (if multiple broad topics, add a row)** | **Broad description (copied and pasted from article)** | **Narrower topic 1 (NT1)** | **NT1 description (copied and pasted from article)** | **Narrower topic 2 (NT2)** | **NT2 description (copied and pasted from article)** |
| --- | --- | --- | --- | --- | --- | --- | --- | --- | --- | --- |
| Rice 2017 | Journal article | Updating systematic reviews, article testing to see if using PubMed Clinical Queries combined with the PubMed similar article feature is as good as the old-fashioned search methods for updating reviews | The objective of the study was to test the overall effectiveness of a simplified systematic search strategy (SSS) for updating systematic reviews | SR | Selecting sources to search | An SSS in MEDLINE limited using clinical queries combined with PubMed’s “similar articles” search | Filters: PubMed Clinical Queries | A search algorithm using a simplified subject search developed by an experienced librarian and limited using MEDLINE’S clinical queries | PubMed’s similar article feature | PubMed’s related articles search (now “similar articles”) |
| University of Michigan Library 2018 | Web guide | Doing an SR, a general web guide on the steps of an SR, with some extra focus on the creating a systematic search strategy section | Information on how to conduct systematic reviews in the health sciences | SR | Designing a search string | A well-constructed systematic search strategy is the core of your systematic review and will be reported on in the methods section of your paper | Using both keywords and subject (MeSH) terms | Both types of search terms are useful, and both should be used in your search. | Using study type filters | In constructing your search by using specific search filters |
| Kugley, 2016 | Guide from organization | Doing a Campbell review, the information retrieval guide from the Campbell Collaboration, their equivalent of chapter 6 from Cochrane | ...considerations on the information retrieval process... in the planning and conduct of Campbell systematic reviews | SR | Selecting sources to search | Objective and reproducible search of a range of sources to identify as many relevant studies as possible | Searching broadly | Search of a range of sources | Search for non-English studies | Citations published in many languages |

SR=systematic review, HTA= health technology assessment, CPG= clinical practice guideline, LR= literature review, MeSH=Medical Subject Headings.
