# Supplementary material for: The decisions and processes involved in a systematic search strategy: a hierarchical framework
Source: J Med Libr Assoc. 2021 Apr 1;109(2):201–11. doi: 10.5195/jmla.2021.1086 (PMC8270345; doi:10.5195/jmla.2021.1086)
Supplement: Supplementary file 3 — Appendix C: Documents used to create the hierarchical framework [file jmla-109-2-201-s03.docx]

# The decisions and processes involved in a systematic search strategy: a hierarchical framework

## Justin Michael Clark; Elaine Beller; Paul Glasziou; Sharon Sanders

### APPENDIX C

### Documents used to create the hierarchical framework

Aagaard T, Lund H, Juhl C. Optimizing literature search in systematic reviews - are MEDLINE, EMBASE and CENTRAL enough for identifying effect studies within the area of musculoskeletal disorders? BMC Med Res Methodol. 2016 Nov 22;16(1):161.

Adams AL. Planning search strategies for maximum retrieval from bibliographic databases. Online Rev. 1979;3(4):373–9.

Adams J, Hillier-Brown FC, Moore HJ, Lake AA, Araujo-Soares V, White M, Summerbell C. Searching and synthesising ‘grey literature’ and ‘grey information’ in public health: critical reflections on three case studies. Syst Rev. 2016 Sep 29;5(1):164.

Alca Alcaide Jiménez JF, Imaz Iglesia I, González Enríquez J, Bravo Toledo R, Conde Olasagasti JL. [Searching for evidence. a compilation of useful resources for health technology assessment]. Med Clin (Barc). 2000;114(suppl 2):105–10.

Alexanderson K, Norlund A; Swedish Council on Technology Assessment in Health Care (SBU). Chapter 2. Methods used for the systematic literature search and for the review of relevance, quality, and evidence of studies. Scand J Public Health Suppl. 2004;63:31–5.

Alpi KM, Stringer E, Devoe RS, Stoskopf M. Clinical and research searching on the wild side: exploring the veterinary literature. J Med Libr Assoc. 2009 Jul;97(3):169–77. DOI: <http://dx.doi.org/10.3163/1536-5050.97.3.005>.

Aromataris E, Munn Z, eds. Joanna Briggs Institute reviewer’s manual. Joanna Briggs Institute; 2017.

Atsawawaranunt K, Adams CE, Roberts S. Searching for randomised controlled trials and clinical controlled trials in Thai online bibliographical biomedical databases. Health Inf Libr J. 2011 Mar;28(1):68–76.

Avenell A, Handoll HH, Grant AM. Lessons for search strategies from a systematic review, in the Cochrane Library, of nutritional supplementation trials in patients after hip fracture. Am J Clin Nutr. 2001 Mar;73(3):505–10.

Bachmann LM, Coray R, Estermann P, Ter Riet G. Identifying diagnostic studies in MEDLINE: reducing the number needed to read. J Am Med Inform Assoc. 2002 Nov–Dec;9(6):653–8.

Badia G. Google Scholar out-performs many subscription databases when keyword searching. Evidence Based Libr Inf Pract. 2010;5(3):39–41.

Bak G, Mierzwinski-Urban M, Fitzsimmons H, Morrison A, Maden-Jenkins M. A pragmatic critical appraisal instrument for search filters: introducing the CADTH CAI. Health Inf Libr J. 2009 Sep26(3):211–9.

Barroso J, Gollop CJ, Sandelowski M, Meynell J, Pearce PF, Collins LJ. The challenges of searching for and retrieving qualitative studies. West J Nurs Res. 2003 Mar;25(2):153–78.

Basch R. The seven deadly sins of full-text searching. Database. 1989;12(4):15–23.

Baudard M, Yavchitz A, Ravaud P, Perrodeau E, Boutron I. Impact of searching clinical trial registries in systematic reviews of pharmaceutical treatments: methodological systematic review and reanalysis of meta-analyses. BMJ. 2017 Feb 17;356:j448.

Beall J. The weaknesses of full-text searching. J Acad Librariansh. 2008 Sep;34(5):438–44.

Beall J, Kafadar K. Measuring the extent of the synonym problem in full-text searching. Evidence Based Libr Inf Pract. 2008;3(4):18–33.

Birch DW, Eady A, Robertson D, De Pauw S, Tandan V; Evidence-Based Surgery Working Group. Users’ guide to the surgical literature: how to perform a literature search. Can J Surg. 2003 Apr;46(2):136–41.

Blackhall K, Ker K. Searching for studies for inclusion in Cochrane Systematic Reviews on injury prevention. Inj Prev. 2008 Apr;14(2):137–8.

Blümle A, Antes G, Diener MK. [Hand searching for controlled clinical trials in German surgical journals. a contribution to evidence-based surgery]. Chirurg. 2007 Nov;78(11):1052–7.

Boeker M, Vach W, Motschall E. Semantically equivalent PubMed and Ovid-MEDLINE queries: different retrieval results because of database subset inclusion. J Clin Epidemiol. 2012;65(8):915–6.

Boeker M, Vach W, Motschall E. Google Scholar as replacement for systematic literature searches: good relative recall and precision are not enough. BMC Med Res Methodol. 2013 Oct 26;13:131.

Booth A. “Brimful of STARLITE”: toward standards for reporting literature searches. J Med Libr Assoc. 2006 Oct;94(4):421–9, e205. (Available from: <<https://www.ncbi.nlm.nih.gov/pmc/articles/PMC1629442/>>. [cited 22 Dec 2020].)

Bramer WM, Rethlefsen ML, Mast F, Kleijnen J. Evaluation of a new method for librarian-mediated literature searches for systematic reviews. Res Synth Methods. 2018 Dec;9(4):510–20.

Briscoe S. Web searching for systematic reviews: a case study of reporting standards in the UK Health Technology Assessment programme. BMC Res Notes. 2015 Apr 16;8:153.

Centre for Reviews and Dissemination. Systematic reviews: CRD’s guidance for undertaking reviews in healthcare. University of York, UK: Centre for Reviews and Dissemination; 2009.

Cohen JF, Korevaar DA, Wang J, Spijker R, Bossuyt PM. Should we search Chinese biomedical databases when performing systematic reviews? Syst Rev. 2015 Mar 6;4:23.

Craven J, Levay P. Recording database searches for systematic reviews - what is the value of adding a narrative to peer-review checklists? a case study of NICE interventional procedures guidance. Evidence Based Libr Inf Pract. 2011;6:72–87.

Crumley E, Bhatnagar N, Stobart K. Peer reviewing comprehensive search strategies in hemophilia and von Willebrand disease. J Can Health Libr Assoc. 2004;25(4):113–6.

Cullen R, Mason D. Quality assurance in health sciences literature searching: applying the ISO 9000 quality standard. Health Libr Rev. 1995 Sep;12(3):173–89.

Day D, Furlan A, Irvin E, Bombardier C. Simplified search strategies were effective in identifying clinical trials of pharmaceuticals and physical modalities. J Clin Epidemiol. 2005 Sep;58(9):874–81.

de Freitas AE, Herbert RD, Latimer J, Ferreira PH. Searching the LILACS database for Portuguese- and Spanish-language randomized trials in physiotherapy was difficult. J Clin Epidemiol. 2005 Mar;58(3):233–7.

de Vries RB, Hooijmans CR, Tillema A, Leenaars M, Ritskes-Hoitinga M. Updated version of the Embase search filter for animal studies. Lab Anim. 2014 Jan;48(1):88.

DeLuca JB, Mullins MM, Lyles CM, Crepaz N, Kay L, Thadiparthi S. Developing a comprehensive systematic search strategy for evidence based systematic reviews. Evidence Based Libr Inf Pract. 2008;3(1):3–32.

Edith Cowan University Library. Systematic reviews: systematic review process [Internet]. The Library; 2018 [cited 22 Dec 2020]. <<http://ecu.au.libguides.com/c.php?g=410585&p=2797692>>.

Fitzgibbons M, Meert D. Are bibliographic management software search interfaces reliable?: a comparison between search results obtained using database interfaces and the EndNote online search function. J Acad Librariansh. 2010 Mar;36(2):144–50.

Ganshorn H. Translation of hedges in medical databases to other platforms’ syntax may cause significantly different search results. Evidence Based Libr Inf Pract. 2011;6(2):55–8.

Goetz T, von der Lieth CW. PubFinder: a tool for improving retrieval rate of relevant PubMed abstracts. Nucleic Acids Res. 2005 Jul 1;33(web server issue):W774–8.

Golder S, Loke Y, McIntosh HM. Poor reporting and inadequate searches were apparent in systematic reviews of adverse effects. J Clin Epidemiol. 2008 May 61(5):440–8.

Hariri N, Ravandi SN. Comparing the precision of information retrieval of MeSH-controlled vocabulary search method and a visual method in the MEDLINE medical database. Electron Physician. 2014 May 10;6(2):832–7.

Hawkins N, Scott DA, Woods B. How far do you go? efficient searching for indirect evidence. Med Decis Making. 2009 May–Jun;29(3):273–81.

Iansavichene AE, Sampson M, McGowan J, Ajiferuke IS. Should systematic reviewers search for randomized, controlled trials published as letters? Ann Intern Med. 2008 May 6;148(9):714–5.

Koffel JB. Use of recommended search strategies in systematic reviews and the impact of librarian involvement: a cross-sectional survey of recent authors. PLoS One. 2015 May 4;10(5):e0125931.

Kugley S, Wade A, Thomas J, Mahood Q, Jørgensen AMK, Hammerstrøm K, Sathe N. Searching for studies: a guide to information retrieval for Campbell [Internet]. Campbell Systematic Reviews; 2016 [cited 22 Dec 2020]. <<http://www.campbellcollaboration.org/images/Campbell_Methods_Guides_Information_Retrieval.pdf>>.

Lefebvre C, Manheimer E, Glanville J. Searching for studies. Cochrane handbook for systematic reviews of interventions. John Wiley & Sons; 2008. p. 95–150.

Lui S, Smith EJ, Terplan M. Heterogeneity in search strategies among Cochrane acupuncture reviews: is there room for improvement? Acupunct Med. 2010 Sep;28(3):149–53.

Lyon JA, Garcia-Milian R, Norton HF, Tennant MR. The use of Research Electronic Data Capture (REDCap) software to create a database of librarian-mediated literature searches. Med Ref Serv Q. 2014;33(3):241–52.

McGowan J, Sampson M, Lefebvre C. An evidence based checklist for the Peer review of electronic search strategies (PRESS EBC). Evidence Based Libr Inf Pract. 2010;5(1):149–54.

Morris RT, Holtum EA, Curry DS. Being there: the effect of the user’s presence on MEDLINE search results. Bull Med Libr Assoc. 1982 Jul;70(3):298–304. (Available from: <<https://www.ncbi.nlm.nih.gov/pmc/articles/PMC226712/>>. [cited 22 Dec 2020].)

National Institute for Health and Care Excellence (NICE). Developing NICE guidelines: the manual. The Institute; 2017.

Newcastle University Library. Systematic reviews [Internet]. The Library; 2018 [cited 22 Dec 2020]. <<https://libguides.ncl.ac.uk/systematicreviews>>.

Notess GR. Intricacies of phrase searching. Online Searcher. 2014 Nov/Dec;38(6):65–7.

O’Mara-Eves A, Brunton G, McDaid D, Kavanagh J, Oliver S, Thomas J. Techniques for identifying cross-disciplinary and ‘hard-to-detect’ evidence for systematic review. Res Synth Methods. 2014 Mar;5(1):50–9.

Ormstad SS, Isojarvi J. Information retrieval for health technology assessment: standardization of search methods. Int J Technol Assess Health Care. 2010 Oct;26(4):359–61.

Oxford Library. Systematic reviews: searching for primary studies [Internet]. The Library; 2017 [cited 22 Dec 2020]. <<https://libguides.bodleian.ox.ac.uk/systematic-reviews/searchingforprimarystudies>>.

Pasterczyk CE. Russian transliteration variations for searchers. Database. 1985 Feb;8(1):68–75.

Petrova M, Sutcliffe P, Fulford KW, Dale J. Search terms and a validated brief search filter to retrieve publications on health-related values in MEDLINE: a word frequency analysis study. J Am Med Inform Assoc. 2012 May–Jun19(3):479–88.

Pilkington K, Boshnakova A, Clarke M, Richardson J. No language restrictions in database searches: what does this really mean? J Altern Complement Med. 2005 Feb;11(1):205–7.

Quint B. Format searching. a technique that helps new end-user searchers cover huge databases with simplified search strategies. Online. 1985 May;9(3):23–8.

Rice M, Ali MU, Fitzpatrick-Lewis D, Kenny M, Raina P, Sherifali D. Testing the effectiveness of simplified search strategies for updating systematic reviews. J Clin Epidemiol. 2017 Aug;88:148–53.

Royle P, Waugh N. Should systematic reviews include searches for published errata? Health Inf Libr J. 2004 Mar;21(1):14–20.

Saleh AA, Ratajeski MA, Bertolet M. Grey literature searching for health sciences systematic reviews: a prospective study of time spent and resources utilized. Evidence Based Libr Inf Pract. 2014;9(3):28.

Sampson M, McGowan J. Inquisitio validus Index Medicus: a simple method of validating MEDLINE systematic review searches. Res Synth Methods. 2011 Jun;2(2):103–9.

Sampson M, Shojania KG, McGowan J, Daniel R, Rader T, Iansavichene AE, Ji J, Ansari MT, Moher D. Surveillance search techniques identified the need to update systematic reviews. J Clin Epidemiol. 2008 Aug;61(8):755–62.

Siddaway A. What is a systematic literature review and how do I do one? University of Stirling; 2014.

Sladek RM, Tieman J, Currow DC. Searchers be aware: limiting PubMed searches to ‘humans’ loses more than you think. Intern Med J. 2010 Jan;40(1):88–9.

Southern Cross University Library. Systematic reviews [Internet]. The Library; 2018 [cited 22 Dec 2020]. <<http://libguides.scu.edu.au/systrev>>.

Stamm T, Hohoff A. Nonlinear behavior of search strategies for identifying relevant orthodontic articles. Angle Orthod 2004 Jun;74(3):316–8.

Tanon AA, Champagne F, Contandriopoulos AP, Pomey MP, Vadeboncoeur A, Nguyen H. Patient safety and systematic reviews: finding papers indexed in MEDLINE, EMBASE and CINAHL. Qual Saf Health Care. 2010 Oct;19(5):452–61.

Taylor B, Wylie E, Dempster M, Donnelly M. Systematically retrieving research: a case study evaluating seven databases. Res Soc Work Pract. 2007;17(6):697–706.

University of Michigan Library. Systematic reviews: information on how to conduct systematic reviews in the health sciences: creating a systematic search strategy [Internet]. The Library; 2018 [cited 22 Dec 2020]. <<http://guides.lib.umich.edu/c.php?g=283340&p=2126706>>.

Wang JF, Li ZR, Cai CZ, Chen YZ. Assessment of approximate string matching in a biomedical text retrieval problem. Comput Biol Med. 2005 Oct;35(8):717–24.

Wilczynski NL, McKibbon KA, Haynes RB. Search filter precision can be improved by NOTing out irrelevant content. AMIA Annu Symp Proc. 2011;1506–13.

Younger P, Boddy K. When is a search not a search? a comparison of searching the AMED complementary health database via EBSCOhost, OVID and DIALOG. Health Inf Libr J. 2009 Jun;26(2):126–35.
