# Supplementary material for: The decisions and processes involved in a systematic search strategy: a hierarchical framework
Source: J Med Libr Assoc. 2021 Apr 1;109(2):201–11. doi: 10.5195/jmla.2021.1086 (PMC8270345; doi:10.5195/jmla.2021.1086)
Supplement: Supplementary file 4 — Appendix D: Studies used to test the hierarchical framework [file jmla-109-2-201-s04.docx]

# The decisions and processes involved in a systematic search strategy: a hierarchical framework

## Justin Michael Clark; Elaine Beller; Paul Glasziou; Sharon Sanders

### APPENDIX D

### Studies used to test the hierarchical framework

1. Ayiku L, Levay P, Hudson T, Craven J, Finnegan A, Adams R, Barrett E. The Embase UK filter: validation of a geographic search filter to retrieve research about the UK from OVID Embase. Health Inf Libr J. 2019 Jun;36(2):121–33.
2. Burns CS, Shapiro II RM, Nix T, Huber JT. Search results outliers among MEDLINE platforms. J Med Libr Assoc. 2019 Jul;107(3):364–73. DOI: <http://dx.doi.org/10.5195/jmla.2019.622>.
3. Cooper C, Varley-Campbell J, Carter P. Established search filters may miss studies when identifying randomized controlled trials. J Clin Epidemiol. 2019 Aug;112:12–9.
4. Curkovic M. The implications of using Internet search engines in structured scientific reviews. Sci Eng Eth. 2019;25(2):645–6.
5. Damarell RA, May N, Hammond S, Sladek RM, Tieman JJ. Topic search filters: a systematic scoping review. Health Inf Libr J. 2019 Mar;36(1):4–40.
6. Frandsen TF, Gildberg FA, Tingleff EB. Searching for qualitative health research required several databases and alternative search strategies: a study of coverage in bibliographic databases. J Clin Epidemiol. 2019 Oct;114:118–24.
7. Glanville J, Dooley G, Wisniewski S, Foxlee R, Noel-Storr A. Development of a search filter to identify reports of controlled clinical trials within CINAHL Plus. Health Inf Libr J. 2019 Mar;36(1):73–90.
8. Glusker A. Naming specific adverse effects improves relative recall for search filters identifying literature on surgical interventions in MEDLINE and Embase. Evid Based Libr Inf Pract. 2019;14(1):65–7.
9. Golder S, Farrah K, Mierzwinski-Urban M, Wright K, Loke YK. The development of search filters for adverse effects of medical devices in MEDLINE and Embase. Health Inf Libr J. 2019 Sep;36(3):244–63.
10. Gorayeb RP, Forjaz MJ, Ferreira AG, Duarte GNS, Machado T, Ferreira JJ. Electronic search strategies fail to identify randomized controlled trials (RCTs) in neurosurgery. Clin Neurol Neurosurg. 2019 Sep;184:105446.
11. Hosking J, Macmillan A, Jones R, Ameratunga S, Woodward A. Searching for health equity: validation of a search filter for ethnic and socioeconomic inequalities in transport. Syst Rev. 2019 Apr 11;8(1):94.
12. Ioerger M, Flanders RM, Goss KD, Turk MA. Developing a systematic search strategy related to people with disability: a brief report testing the utility of proposed disability search terms in a search about opioid use. Disabil Health J. 2019 Apr;12(2):318–22.
13. Neilson C, Mê-Linh L. A failed attempt at developing a search filter for systematic review methodology articles in Ovid Embase. J Med Libr Assoc. 2019 Apr;107(2):203–9. DOI: <http://dx.doi.org/10.5195/jmla.2019.519>.
14. Pereira RA, Puga M, Atallah AN, Macedo EC, Macedo CR. Lilacs systematic search strategy for systematic reviews of diagnostic test accuracy studies. Health Inf Libr J. 2019 Sep;36(3):223–43.
15. Powell K. Searching by grant number: comparison of funding acknowledgments in NIH RePORTER, PubMed, and Web of Science. J Med Libr Assoc. 2019 Apr;107(2):172–8. DOI: <http://dx.doi.org/10.5195/jmla.2019.554>.
16. Rastegarfar B, Ardalan A, Nejat S, Keshtkar A, Moradian MJ. A productive proposed search syntax for health disaster preparedness research. Bull Emerg Trauma. 2019 Apr;7(2):93–8.
17. Sperr Jr. EV. Mind the gap: identifying what is missed when searching only the broad scope with clinical queries. J Med Libr Assoc. 2019 Jul;107(3):333–40. DOI: <http://dx.doi.org/10.5195/jmla.2019.589>.
18. Tudor Car L, Li L, Smith H, Atun R. Cochrane review: search strategies to identify observational studies in MEDLINE and EMBASE. J Evid Based Med. 2019 Aug;12(3):225–6.
19. Wanner A, Baumann N. Design and implementation of a tool for conversion of search strategies between PubMed and Ovid MEDLINE. Res Synth Methods. 2019 Jun;10(2):154–60.
20. Yu Y, Xu E, Xia E, Huang H, Hao B, Zhang S. A method to accelerate and visualize iterative clinical paper searching. Stud Health Technol Inform. 2019 Aug 21;264:1332–6.
