# Supplementary material for: The decisions and processes involved in a systematic search strategy: a hierarchical framework
Source: J Med Libr Assoc. 2021 Apr 1;109(2):201–11. doi: 10.5195/jmla.2021.1086 (PMC8270345; doi:10.5195/jmla.2021.1086)
Supplement: Supplementary file 5 — Appendix E: Decisions or processes of studies on searching identified in 2019 [file jmla-109-2-201-s05.docx]

# The decisions and processes involved in a systematic search strategy: a hierarchical framework

## Justin Michael Clark; Elaine Beller; Paul Glasziou; Sharon Sanders

### APPENDIX E

### Decisions or processes of studies on searching identified in 2019

| **Author** | **Year** | **Entry no.** | **Decision or process** |
| --- | --- | --- | --- |
| Frandsen | 2019 | 2.2.1 | Searching bibliographic databases |
| Curkovic | 2019 | 2.2.6 | Searching the Internet |
| Ayiku | 2019 | 3.2.15 | Using validated search filters |
| Cooper | 2019 | 3.2.15 | Using validated search filters |
| Damarell | 2019 | 3.2.15 | Using validated search filters |
| Glanville | 2019 | 3.2.15 | Using validated search filters |
| Glusker | 2019 | 3.2.15 | Using validated search filters |
| Golder | 2019 | 3.2.15 | Using validated search filters |
| Gorayeb | 2019 | 3.2.15 | Using validated search filters |
| Hosking | 2019 | 3.2.15 | Using validated search filters |
| Neilson | 2019 | 3.2.15 | Using validated search filters |
| Rastegarfar | 2019 | 3.2.15 | Using validated search filters |
| Tudor | 2019 | 3.2.15 | Using validated search filters |
| Ioerger | 2019 | 3.2.4 | Using broad or focused concepts |
| Sperr | 2019 | 3.2.4 | Using broad or focused concepts |
| Burns | 2019 | 3.2.8 | Selecting a user interface |
| Powell | 2019 | 3.4.10 | Searching fields |
| Pereira | 2019 | 3.6.4 | Modifying for other databases |
| Wanner | 2019 | 3.6.4 | Modifying for other databases |
| Yu | 2019 | 4.1.1 | Using a related articles feature |
