# Supplementary material for: The decisions and processes involved in a systematic search strategy: a hierarchical framework
Source: J Med Libr Assoc. 2021 Apr 1;109(2):201–11. doi: 10.5195/jmla.2021.1086 (PMC8270345; doi:10.5195/jmla.2021.1086)
Supplement: Supplementary file 6 — Appendix F: Hierarchical framework of decisions or processes that can be used to conduct a systematic search with scope notes [file jmla-109-2-201-s06.docx]

# The decisions and processes involved in a systematic search strategy: a hierarchical framework

## Justin Michael Clark; Elaine Beller; Paul Glasziou; Sharon Sanders

### APPENDIX F

### Hierarchical framework of decisions or processes that can be used to conduct a systematic search with scope notes

| **Topic** | **Category** | **Decision or process** | **Scope note** |
| --- | --- | --- | --- |
| 1 Skill of the searcher | | | The skills, experience, and knowledge the person or people designing and conducting the search has in searching for information |
|  | 1.1 Searcher role | | The role, or job, the person or people designing and conducting the search currently or has previously had |
|  |  | 1.1.1 Authors conducting the search | Having authors or other non-search specialists conduct the search |
|  |  | 1.1.2 Cochrane information specialist conducting the search | Having a Cochrane-trained information specialist conduct the search |
|  |  | 1.1.3 Information specialist conducting the search | Having someone who specializes in searching for information for systematic reviews (or other form of evidence synthesis) conduct the search |
|  |  | 1.1.4 Health librarian conducting the search | Having a health librarian conduct the search |
|  |  | 1.1.5 General librarian conducting the search | Having a librarian who specializes in a non-health domain conduct the search |
|  | 1.2 Searcher experience | | The experience or knowledge the person or people designing and conducting the search has in areas which could impact on the search, such as knowledge of the topic, systematic reviews, or searching the literature |
|  |  | 1.2.1 Systematic review experience | Having someone with experience of conducting a previous systematic review but not necessarily the search component, conduct the search |
|  |  | 1.2.2 Experience with the topic | Having someone with knowledge of the topic conduct the search |
|  |  | 1.2.3 Systematic searching experience | Having someone with experience of searching for studies for systematic reviews conduct the search |
|  |  | 1.2.4 General literature search experience | Having someone with experience of searching for information, this could be doing searching for a clinical answer service or helping students with searching for assignments conduct the search |
|  |  | 1.2.5 Training in searching | Having search training provided to the person conducting the search |
| 2 Selecting information to identify | | | Deciding where the information needed to answer the SR question is likely to be found |
|  | 2.1 Selecting publication formats | | Deciding which publication format is likely to contain the information needed to answer the SR question is likely to be reported (e.g., if including journal articles only is acceptable or if books, government reports, or data sets might be required) |
|  |  | 2.1.1 Searching for journal articles | Searching for articles published in journals |
|  |  | 2.1.2 Searching for ongoing studies | Searching for studies that have started, which have normally been registered, but that have not yet been completed |
|  |  | 2.1.3 Searching for nongovernment reports | Searching for reports published by organizations that are not affiliated to a government (e.g., from organization websites) |
|  |  | 2.1.4 Searching for books or book chapters | Searching for books or book chapters |
|  |  | 2.1.5 Searching for conference proceedings | Searching for conference proceedings |
|  |  | 2.1.6 Searching for dissertations | Searching for dissertations or theses |
|  |  | 2.1.7 Searching for correspondence | Searching for letters to the editor |
|  |  | 2.1.8 Searching for electronic publications ahead of print | Searching for epubs ahead of print |
|  |  | 2.1.9 Searching for language-specific information | Searching for information in specific languages |
|  |  | 2.1.10 Searching for government reports | Searching for reports published by government departments |
|  |  | 2.1.11 Searching for unpublished work | Searching for research that has not been published in a journal, book, or dissertation |
|  |  | 2.1.12 Searching for errata or corrections | Searching for notifications of errata or corrected publications |
|  |  | 2.1.13 Excluding predatory journals | Searching in a way that excludes predatory journals, such as using curated databases like PubMed, or excluding based on title of journal |
|  | 2.2 Selecting electronic sources to search | | Deciding which electronic sources of information should be searched using a search string |
|  |  | 2.2.1 Searching bibliographic databases | Searching databases that contain titles and abstracts of journal articles to search, taking account of their scope (e.g., PubMed for health generally, CINAHL for nursing, LILACS for South American material) |
|  |  | 2.2.2 Searching full-text databases | Searching databases that contain the full texts of journal articles (and, sometimes, other content such as book chapters) to search (e.g., PubMed Central, ScienceDirect) |
|  |  | 2.2.3 Searching specialized registers | Searching registers that focus on a specific area (e.g., the Cochrane Neonatal specialized register, which only contains neonatal articles) |
|  |  | 2.2.4 Searching Google Scholar | Searching Google Scholar |
|  |  | 2.2.5 Searching trial registries | Searching clinical trial registers (e.g., Clinicaltrials.gov or the World Health Organization [WHO] International Clinical Trials Registry Platform [ICTRP]) |
|  |  | 2.2.6 Searching the Internet | Searching the Internet generally (e.g., using a search engine such as Google) |
|  |  | 2.2.7 Searching specific websites | Searching specific types of websites (e.g., journals [for their full-text articles], conferences, government, or organization websites) |
|  |  | 2.2.8 Searching social media platforms | Searching social media platforms, (e.g., Twitter) |
|  |  | 2.2.9 Costs of searching | Deciding how much to spend on searching (e.g., paying for access to databases that are not freely available) |
| 3 Searching the literature electronically | | | Entering a search string into an electronic source, then running that search string |
|  | 3.1 Obtaining a development article set | | Ways to obtain a set of articles to develop a search string, this can be used to help design the search string or to validate the search string, also known as a total set, test set, or comparator set |
|  |  | 3.1.1 Obtaining a development set from systematic reviews | Looking through systematic reviews on a similar or the same topic for articles that will help build and/or test the search string |
|  |  | 3.1.2 Obtaining a development set from experts | Asking people with knowledge of the topic if they know of articles that will help build and/or test the search string |
|  |  | 3.1.3 Obtaining a development set from a scoping search | Running a highly focused search to identify a sample set of articles that will help build and/or test the search string |
|  |  | 3.1.4 Obtaining a development set from a citation analysis | Doing a backward and/or forward citation analysis to find a sample set of articles that will help build and/or test the search string |
|  | 3.2 Conceptualizing the search string | | Decide how the search string fits together with the aim of identifying all relevant studies while minimizing the number of irrelevant studies |
|  |  | 3.2.1 Selecting search concepts | Choosing which concepts to include in the search without any reference to a structured format that labels the concepts (e.g., one concept may be the disease [pneumonia] and another concept may be the treatment [antibiotics] another concept may be the fact the treatment is) |
|  |  | 3.2.2 Selecting concepts from the systematic review’s patient, intervention, comparison, outcome (PICO) | Using the PICO format of the systematic review as the concepts in the search string |
|  |  | 3.2.3 Selecting concepts using a structured format | Using a structured format, other than the PICO format, to select the concepts to add to the search string |
|  |  | 3.2.4 Using broad or focused concepts | Deciding whether to use broader, more general, or more focused, less general, concepts in the search string (e.g., searching for cancer [broad] or searching for bladder cancer [focused]) |
|  |  | 3.2.5 Importance of recall | Deciding to maximize the probability of finding all eligible studies |
|  |  | 3.2.6 Importance of precision | Deciding to minimize the number of ineligible studies found |
|  |  | 3.2.7 Searching the full text | Deciding whether to run the search through the full text of articles, rather than just the title, abstracts, and keywords |
|  |  | 3.2.8 Selecting a user interface | Selecting the user interface to use for the database (e.g., PubMed, Ovid MEDLINE, or EBSCO MEDLINE) |
|  |  | 3.2.9 Database used for designing primary search string | Deciding which database (e.g., PubMed or Embase) to use for the initial search and preparing the search string accordingly |
|  |  | 3.2.10 Searching for older studies | Deciding on the approach to search for older articles that might not have an abstract in the database |
|  |  | 3.2.11 Ordering of search words | Deciding on the order for entering the search word ( e.g., vaccine AND child, or child AND vaccine) |
|  |  | 3.2.12 Using “AND” or “OR” | Using “AND” and “OR” in the search string |
|  |  | 3.2.13 Using “Adjacency” | Deciding whether to use adjacency in your search string |
|  |  | 3.2.14 Using “NOT” | Deciding whether to explicitly exclude articles because they contain certain words (e.g., NOT animals) |
|  |  | 3.2.15 Using validated search filters | Using existing filters that have been tested and validated to find studies on specific topics (e.g., for certain study type like RCYS, or for children or adults) |
|  |  | 3.2.16 Using non-validated search strings | Using search strings from an existing SR on the same or a similar topic |
|  | 3.3 Selecting search words and terms | | The methods used to select the words and index terms that will be used in the search string to search the electronic sources |
|  |  | 3.3.1 Selecting index terms | Choosing eligible index terms from the source to be searched (e.g., MeSH terms for PubMed), these are often referred to as “subject terms,” “controlled terms,” “controlled vocabulary terms,” or “controlled language” |
|  |  | 3.3.2 Searcher selecting search words | The person designing the search string uses their personal knowledge and opinion to select the words to use in the search string, also referred to as “free text terms,” “keywords,” or “natural language” |
|  |  | 3.3.3 Selecting search words from a development set | Looking through articles in a development set for words to add to a search string that will help the search string identify the maximum number of relevant studies |
|  |  | 3.3.4 Selecting search words from word frequency analysis | Selecting search words based upon the number of times those words appear in a development set of articles |
|  |  | 3.3.5 Obtaining search words from experts | Asking people with a good understanding of the topic to suggest words to use in the search string |
|  |  | 3.3.6 Selecting search words from similar articles | Selecting words from articles on a similar but not identical topic to put into a search string |
|  |  | 3.3.7 Using synonyms | Using synonyms for the concepts in the search string |
|  |  | 3.3.8 Using alternate spellings | Using English and/or American spellings, common misspellings or alternative spelling of words (e.g., cesarian, caesarean, cesarean) |
|  |  | 3.3.9 Using words in other languages | Choosing search words from different languages (either in those languages or English language words that originate from other languages [e.g., “aleatory”]) |
|  | 3.4 Using database search commands | | Using features and commands available in the database being searched |
|  |  | 3.4.1 Exploding index terms | Using the explode feature on an index term to look for all its narrower index terms |
|  |  | 3.4.2 Focusing index terms | Using the focus command to retrieve only those studies in which the index term is considered to be the primary focus of the study |
|  |  | 3.4.3 Using search words and index terms | Using both search words and index terms in a search string |
|  |  | 3.4.4 Using search words alone | Using search words alone in the search string (e.g., do not use index terms in the search string) |
|  |  | 3.4.5 Using index terms alone | Using index terms alone in the search string (e.g., do not use search words in the search string) |
|  |  | 3.4.6 Using subheadings | Using index term subheadings, either attached to the index term or alone in a search string (e.g., attaching the subheading "/adverse effects" to the index term "Vaccines" or searching for "/adverse effects" alone to identify studies about the adverse effects of vaccines) |
|  |  | 3.4.7 Using predefined limits | Using the database’s inbuilt limits to restrict the search (e.g., restricting to humans, specific age groups, publication dates, language, particular countries or regions, types of publication, etc.) |
|  |  | 3.4.8 Using wildcards and truncation | Using, for example, "*" to search for plurals or multiple spelling variants |
|  |  | 3.4.9 Using phrase searching | Using phrases, normally shown in quotation marks "" in the search string |
|  |  | 3.4.10 Searching fields | Using a search field command to retrieve only studies where the words appear in specific fields (e.g., searching the title field, the abstract field or the author field) |
|  |  | 3.4.11 Using term mapping | Using the automatic term mapping to index terms or common synonyms done by the database (e.g., a database may also add the index term "Thorax" to a search for the word) |
|  |  | 3.4.12 Using word frequency | Deciding whether to use the number of times a word appears (frequency) as a parameter in the search string (e.g., the “word” must appear at least 3 times in the title, abstract, or index terms to be found by the search string) |
|  | 3.5 Refining a search string | | Refining a preliminary search string |
|  |  | 3.5.1 Refining to improve recall | Refining the search to increase the probability of finding eligible articles |
|  |  | 3.5.2 Refining to improving precision | Refining the search to decrease the probability of finding ineligible articles |
|  |  | 3.5.3 Using validation articles | Determining if the search retrieved articles from a test set of known eligible articles |
|  |  | 3.5.4 Updating search words and index terms | Adding or changing search words or index terms in the search string because of changes in terminology |
|  |  | 3.5.5 Using words in other languages | Testing each word used in the search individually (perhaps including each synonym) |
|  |  | 3.5.6 Discussing the search string with experts | Refining the search through discussions with experts (including coauthors) |
|  |  | 3.5.7 Peer reviewing the search string | Having a person with some knowledge of searching provide feedback on the search string, this can be done with or without a checklist |
|  |  | 3.5.8 Using spell checking on the search string | Using an electronic dictionary to check the spelling of the words in the search string |
|  |  | 3.5.9 Finalizing the search string | Determining when the search string meets its purpose and can be run across all databases |
|  | 3.6 Running a search string | | Running the designed search string in the appropriate source, normally a database |
|  |  | 3.6.1 Single line searching | Combining all search words and index terms into a search string that can be copied, in its entirety, and run in a database |
|  |  | 3.6.2 Line-by-line searching | Running the search for each search word or index term separately and combining them with multiple AND or OR operators at the end |
|  |  | 3.6.3 Block searching | Running the search string of each concept separately and combining them at the end (e.g., vaccines and its synonyms on 1 line, child and its synonyms on another line) |
|  |  | 3.6.4 Modifying for other databases | Modifying the search string so it can be run in other databases (e.g., by changing index terms manually, modifying the syntax for searching specific fields) |
|  |  | 3.6.5 Modifying for nonbibliographic databases | Modifying the search string so it can be run in a source such as an Internet search engine (e.g., Google) |
| 4 Other ways to identify studies | | | Methods to find eligible studies other than checking the returns from running a search string in an electronic database |
|  | 4.1 Using databases similarity feature | | Using a database feature on a study to retrieve other studies that are similar to that study |
|  |  | 4.1.1 Using a related articles feature | Using a databases content similarity feature with a pool of eligible articles to identify further eligible studies (e.g., the similar articles feature in PubMed) |
|  | 4.2 Contacting people | | Contacting people who may be able to provide information about eligible studies |
|  |  | 4.2.1 Contacting experts | Contacting people with expertise in the topic area for information about eligible studies |
|  |  | 4.2.2 Contacting funders | Contacting agencies that fund research in the topic area for information on eligible studies |
|  |  | 4.2.3 Contacting authors of included studies | Contacting authors of the included studies for additional eligible studies |
|  |  | 4.2.4 Contacting manufacturers | Contacting manufacturers of eligible interventions for information on eligible studies |
|  |  | 4.2.5 Contacting regulatory agencies | Contacting agencies that regulate interventions or research in the topic area for information on eligible studies |
|  |  | 4.2.6 Contacting specialist organizations | Contacting organizations that work in the topic area for information on eligible studies |
|  |  | 4.2.7 Soliciting eligible studies | Setting up a system to allow people to submit eligible studies (e.g., a website with contact details of the authors, title of the systematic review, and list of currently included studies) |
|  | 4.3 Citation analysis | | Looking at articles cited by included studies or that cite an included study |
|  |  | 4.3.1 Selecting a citation database | Selecting which citation database to use (e.g., Scopus, Web of Science, or Google Scholar) |
|  |  | 4.3.2 Conducting a forward citation analysis | Searching for articles, using a citation database, that cited one or more of the included studies, also known as reverse snowballing or citing articles searching |
|  |  | 4.3.3 Conducting a backward citation analysis | Searching for articles, using a citation database, in the reference lists of included studies, also known as snowballing, citation chaining, or cited articles searching |
|  |  | 4.3.4 Conducting a co-citing articles analysis | Identifying articles that share one reference, or more, with the citations in the reference list of the included studies |
|  |  | 4.3.5 Conducting a co-cited articles analysis | Identifying a co-citation network from articles citing the original key articles |
|  |  | 4.3.6 Manually checking reference lists | Checking the references cited by an included study by reading the list in the article, as opposed to using a citation database such as Web of Science |
|  |  | 4.3.7 Checking other systematic reviews | Checking the reference lists of systematic reviews on the same or similar topics |
|  |  | 4.3.8 Iterative citation analysis | Conducting a new citation analysis for additional eligible studies found in a previous citation analysis |
|  | 4.4 Hand searching | | Browsing sources that potentially contain eligible studies (e.g., by looking at the table of contents of journals) |
|  |  | 4.4.1 Hand searching journals | Selecting journals known to publish on the topic of the systematic review, then browsing the journals for eligible studies (e.g., screening their table of contents) |
|  |  | 4.4.2 Hand searching websites | Selecting websites that may contain eligible studies, then browsing the websites (e.g., by checking a publications list on a research institutes website) |
|  |  | 4.4.3 Hand searching conference proceedings | Selecting conferences that may have presentations on the topic of the systematic review, then browsing the conference programs |
|  |  | 4.4.4 Hand searching bookshelves and filing cabinets | Selecting books, article reprints, or other items that are stored on shelves or in filing cabinets that may contain eligible studies, then browsing those sources |
|  |  | 4.4.5 Hand searching personal collections | Selecting an expert’s personal collection of studies that may contain eligible studies, then browsing that collection |
|  | 4.5 Nonsystematic study identification | | Methods of finding eligible studies that are not systematic and are hard to replicate |
|  |  | 4.5.1 Browsing the literature | Using any search method in an unstructured and incomplete way to find eligible studies (e.g., running non-documented or free-form searches), also known as berry-picking |
| 5 Updating the systematic review | | | Actions taken when updating a systematic review and, therefore, the systematic search, including deciding when to attempt an update of the review, whether to update the review, and whether to modify the original search. Note: this does not cover decisions about whether the scope of the review should change when it is updated. |
|  | 5.1 Updating the systematic search | | Ways of determining whether new eligible studies are available, for the purpose of deciding whether to update the systematic review |
|  |  | 5.1.1 Receiving table of contents alerts | Receiving tables of contents from journals to check for new eligible studies |
|  |  | 5.1.2 Receiving search alerts | Receiving the results of a search update run automatically by a database or journals |
|  |  | 5.1.3 Periodically rerunning searches | Rerunning the search strings used in the systematic review after a specified period of time |
|  |  | 5.1.4 Surveying the literature | Running regular searches in specific locations |
|  |  | 5.1.5 Monitoring eligible registered trials | Monitoring eligible registered trials to see if they have been completed and their results published |
|  | 5.2 Running the updated systematic search | | Actions done when running the systematic search update |
|  |  | 5.2.1 Modifying original sources | Changing the original sources searched or publication formats searched for |
|  |  | 5.2.2 Using search alerts | Obtaining new search results by an automatic rerunning of searches in electronic databases or journals |
|  |  | 5.2.3 Using date limitations | Running the search update from the date the previous search was run |
|  |  | 5.2.4 Revising the search string | Revising the search string to see if it needs modifying (e.g., checking for changed or new index terms, or synonyms) |
|  |  | 5.2.5 Searching for retractions | Searching for retractions of previously included studies |
